# Folate intake in a Swedish adult population: Food sources and predictive factors

**DOI:** 10.1080/16546628.2017.1328960

**Published:** 2017-06-07

**Authors:** Celia Monteagudo, Henrik Scander, Bente Nilsen, Agneta Yngve

**Affiliations:** ^a^ Department of Food, Nutrition and Dietetics, Uppsala University, Uppsala, Sweden; ^b^ Research Group on Nutrition, Diet and Risk Assessment-AGR255, Department of Nutrition and Food Science, University of Granada, Granada, Spain; ^c^ School of Hospitality, Culinary Arts and Meal Science, Örebro University, Örebro, Sweden

**Keywords:** Dietary habits, Riksmaten study, vegetable consumption, lifestyle habits, demographic differences

## Abstract

**Introduction**: Folate plays an important role in cell metabolism, but international studies show that intake is currently below recommendations, especially among women. The study objective was to identify folate food sources by food group, gender, and age group, and to identify factors influencing folate intake, based on food consumption data for Swedish adults in the 2010–11 Riksmaten study.

**Methods**: The sample included a representative Swedish population aged 18–80 years (*n* = 1657; 56.3% female). Food and nutrient intakes were estimated from self-reported food records during 4 consecutive days. Food consumption was categorized into 26 food groups. Stepwise regression was used to analyze food groups as folate sources for participants. Factors predicting the highest folate intake (third tertile) were determined by logistic regression analysis.

**Results**: Vegetables and pulses represented the most important folate source for all age groups and both genders, especially in women aged 45–64 years (49.7% of total folate intake). The next folate source in importance was dairy products for the youngest group (18–30 years), bread for men, and fruit and berries for women. The likelihood of being in the highest tertile of folate intake (odds ratio = 1.69, 95% confidence interval 1.354–2.104) was higher for men. Influencing factors for folate intake in the highest tertile were low body mass index and high educational level in the men, and high educational level, vegetarian diet, organic product consumption, non-smoking, and alcohol consumption within recommendations in the women.

**Conclusion**: This study describes the folate intake per food group of Swedish adults according to the 2010–11 Riksmaten survey, identifying vegetables and pulses as the most important source. Data obtained on factors related to folate consumption may be useful for the development of specific nutrition education programs to increase the intake of this vitamin in high-risk groups.

## Introduction

The biological activity of folate is related to the production and maintenance of new cells and is therefore in particular demand during periods of rapid cellular growth, such as pregnancy and childhood. Folate intake is also important to prevent cognitive decline in old age and may enhance academic performance in young people [1,2]. The recommended intake for adults is 400 μg/day, with the addition of 200 μg/day during pregnancy and 100 μg/day during breastfeeding [3]. There is evidence that serum folate levels are reduced in smokers and alcohol drinkers [4,5], who may therefore need a higher folate intake.

Current studies show that the folate intake of adults is below recommendations in several countries [6–9]. Food fortification plays an important role in this regard and is mandatory in many American and African countries [10]. Folic-acid fortified foods are also found in Europe (especially cereals), where this fortification is voluntary [11]; however, very few food products are fortified with folic acid in Sweden, where the policy is to recommend folic acid supplements to women planning a pregnancy.

The 2012 European Prospective Investigation into Cancer and Nutrition (EPIC) study in 10 European countries found the mean folate intake to be 307 μg/day for men and 252 μg/day for women [12], while the estimated requirement is 320 μg/day for either gender according to international recommendations [13,14]. Among participating countries, the highest intakes were in the UK, Spain, and France, while the lowest were in Sweden and Norway. The Riksmaten study of the food and nutrient intake in the Swedish population [15], on which the present article is based, found the intake of this vitamin to be higher than in previous years owing to an increase in fruit and vegetable consumption. The highest intake was among physically active participants, women with the highest income levels, and men with physically demanding work. The lowest intake was among smokers and among individuals in households with more than two people. The mean folate intake (259 ± 106 μg/day) was below the recommended level but met the average requirement established by Nordic Nutrition Recommendations [3]. Folate bioavailability depends on the food source and appears to be highest from fruit and vegetables, but limited data are available on this aspect [16]. The food matrix plays a very important role in this regard, and there is incomplete release of cell content from some plant cellular structures [17].

According to the food composition tables for Sweden, the richest sources of folate include liver, legumes, vegetables (mainly green leafy vegetables), fruit, and wholegrain cereals [18]. The 2010–11 Riksmaten survey provided the opportunity to determine the most important folate food sources in the Swedish population, analyzing patterns of consumption (amount, frequency, and food type) by subgroup (age, gender, educational level, and lifestyle). The objective of this study was to identify the most important folate food sources for adult participants in the 2010–11 Riksmaten study by gender and age group and to examine the relationship of demographic and lifestyle factors with their folate intake.

## Material and methods

### Study population

The study sample included a representative Swedish adult population aged 18–80 years from the Riksmaten study (*n* = 5000), previously described in detail by the Swedish National Food Agency [15]. The availability of food intake data from this study was the inclusion criterion for the present investigation (*n* = 1797, 56% women), while exclusion criteria were pregnancy (*n* = 25) and unknown gender (*n* = 115). The final study sample included 1657 participants (56.3% women). The Riksmaten study was approved by the Regional Ethical Review Board of Uppsala, and all participants gave oral informed consent before entering the study.

### Dietetic and nutritional assessment

Food and nutrient intakes were estimated from self-reported food records during 4 consecutive days, described in detail elsewhere [19]. Household measures, numbers of portions (cups, pieces, slices), and grams were used to estimate the amounts consumed [20]. Natural folate sources were studied by categorizing food consumption into 26 food groups, following Ax et al. [19]: fish and shellfish; meat and meat products; eggs; potatoes; vegetables and pulses; fruit and berries; dairy products; cream and crème fraîche; cheese; fast food; pasta, rice, and food grain; bread; cereals; sweet bakery products, sweets (candies), and chocolate; salads (vegetables mixed with cheese, poultry, pasta, bread, nuts, and sauces, among other foods); soups; sauces, dressings, and condiments; substitute products; fats; snacks; nuts and seeds; juice; coffee; tea; soda; and alcoholic beverages.

### Demographic, anthropometric, lifestyle, and food habit covariables

Covariables were considered as dichotomized variables. Cut-off points were 50 years (median value) for age and a body mass index (BMI) of 25 kg/m^2^ (underweight and normal weight vs overweight and obesity) based on self-reported height and weight. Education categories were grouped as higher (university and college) versus lower (3 years of high school, 2 years of high school, elementary school, and illiterate) levels. Food choice was described as vegetarianism (lactovegetarianism, lactovegetarianism including fish and eggs, ovolactovegetarianism, and veganism) versus the eating of all types of food. Special diets considered included those for food intolerance/allergy, weight loss, and the treatment of disease (e.g. diabetes or dyslipidemia). Consumption of organically grown fruit and vegetables was dichotomized as frequent versus occasional or no consumption. Alcohol intake was divided according to the Nordic Nutritional Recommendations [3] between <20 g/day for men or <10 g/day for women and ≥20 g/day or ≥10 g/day, respectively. Smoking was dichotomized as daily versus occasional or no smoking.

### Statistical analysis

Means with standard deviation (SD) were calculated for quantitative variables and frequencies (%) for nominal variables. The Student’s *t*, Pearson’s chi-squared, and analysis of variance (ANOVA) tests were used to study differences by gender and folate intake tertile. Stepwise linear regression [21,22] was performed to analyze folate sources for the Swedish population, with total folate intake (μg/day) as the dependent variable and folate intake (μg/day) from the 26 aforementioned food groups as factors. The distribution of demographics, lifestyle, and dietary data was assessed by tertile of folate intake. Factors predicting the highest folate intake (third tertile) were determined by logistic regression analysis, including the aforementioned covariables (gender, age, BMI, educational level, vegetarian, special diet, consumption of organic fruit and vegetables, nutritional supplementation, smoking, and alcohol intake) in the model. *p* < 0.05 was considered significant. SPSS version 22 (IBM Corp., Armonk, NY, USA) was used for statistical analyses.

## Results

Table 1 exhibits the general characteristics of the study population (Table 1). The mean age was around 50 years and the mean (SD) BMI was within the overweight range. The educational level was high, especially for the women, 45% of whom had studied at university. There was a low frequency of vegetarianism and weight-loss diets, while more than 40% of participants consumed organic fruit and vegetables and almost 50% took nutritional supplements. Overall, 16.2% of participants were smokers, with no difference between the genders (*p* = 0.529); alcohol intake was significantly higher in men than in women (*p* < 0.001).

**Table 1.** General characteristics of the study population.

	Total (*n* = 1657)	Men (*n* = 724)	Women (*n* = 933)	***p***^a^
Age (years)				
Mean	49.61	50.87	48.64	0.007^b^
SD	16.59	16.31	16.74	
Body mass index (kg/m^2^)				
Mean	25.46	26.05	25.00	<0.001^b^
SD	4.34	3.82	4.57	
Educational level (%)				
University	42.5	38.3	45.7	0.002
Non-university	57.5	61.7	54.3	
Vegetarian (%)				
Yes	4.8	3.2	6.1	0.006
Diet (%)				
Yes	5.4	4.9	5.9	0.385
Organic fruit and vegetables (%)				
Yes	42.2	39.9	44.1	0.086
Nutritional supplements (%)				
Yes	49.4	41.3	55.7	<0.001
Alcohol intake (g/day)				
Mean	9.41	11.95	7.46	<0.001^b^
SD	12.25	13.92	10.39	
Smoking (%)				
Yes	16.2	15.5	16.7	0.529

^a^Chi-squared test.

^b^Student’s *t* test.

Table 2 shows the food consumption for men and women by food group. Women reported a higher frequency of all food groups (%), while men reported a significantly higher intake (g/day) of all food groups with the exception of vegetables/beans and fruit/berries, the intake of which was significantly greater in women.

**Table 2.** Percentage of participants who consumed each food group, and grams per day consumed for men and women.

Food groups	Men (*n* = 724)			Women (*n* = 933)			*p*^b^
	%	Mean^a^	SD	%	Mean^a^	SD	
Fish and shellfish/seafood	30.4	71.93	50.72	43.2	55.30	38.81	<0.001
Meat and meat products	42.2	149.54	79.70	53.9	107.07	58.20	<0.001
Eggs	22.0	36.55	29.68	31.5	35.59	28.41	0.626
Potatoes	43.0	139.04	105.07	56.3	79.91	70.70	<0.001
Vegetables and pulses	40.6	124.33	87.50	54.8	142.03	88.69	<0.001
Fruit and berries	34.3	137.67	106.16	51.5	162.62	106.68	<0.001
Milk products	38.0	289.24	191.46	51.3	243.39	156.13	<0.001
Cream and crème fraîche	12.4	20.68	16.72	21.9	18.25	15.81	0.086
Cheese	36.6	28.18	21.83	50.6	26.28	22.39	0.107
Fast food	18.6	119.65	76.74	20.6	89.08	60.73	<0.001
Pasta, rice, and grain	32.8	109.15	71.92	43.5	80.44	57.81	<0.001
Bread	42.8	99.69	51.50	55.2	73.97	39.27	<0.001
Cereals	28.8	78.56	76.20	39.3	63.11	65.36	<0.001
Sweet bakery products and sweets	38.0	80.92	60.55	52.0	66.63	49.70	<0.001
Salads	8.8	73.72	39.99	12.3	68.46	40.21	0.228
Soups	12.6	110.38	59.77	17.4	90.32	56.09	<0.001
Sauces, dressings, and condiments	33.1	47.40	38.18	46.5	38.84	33.93	<0.001
Substitute products	1.7	77.96	63.84	3.3	96.83	83.75	0.299
Fats	35.8	16.02	10.91	47.6	12.02	8.76	<0.001
Snacks	8.7	16.76	13.69	13.7	11.36	12.49	<0.001
Nuts and seeds	8.3	18.46	14.00	17.9	15.04	12.35	0.010
Juice	19.5	143.71	100.07	23.2	120.73	90.29	0.002
Coffee	37.2	441.65	247.99	45.8	406.12	224.67	0.006
Tea	16.8	236.76	186.96	31.4	260.84	237.37	0.116
Soda	23.3	213.05	180.29	28.2	181.55	159.55	0.008
Alcoholic beverages	28.4	273.82	204.87	31.4	176.04	142.77	<0.001

^a^Mean (g/day) intake for individuals reporting consumption of the item.

^b^Student’s *t* test.

Folate sources by age group and gender are displayed in Tables 3 (for women) and 4 (for men). The model for women, which included 18 of the 26 food groups, explained more than 93% of the total folate intake; the main folate source was vegetables and pulses, which provided 49.7% of the total folate intake in women aged 45–64 years. The second folate source was fruit and berries for all age groups with the exception of the youngest women, for whom it was milk products. Salads and pasta, rice, and grains only entered the model for women aged 31–44 years. In accordance with Ax et al. [19], salads were not considered in the vegetables/bean group because they often include not only vegetables (e.g. lettuce and cucumber) but also non-vegetable ingredients such as cheese, poultry, pasta, bread, and sauces, among others. The model for men, which included 20 of the 26 food groups, explained 93% of the total folate intake. As for the women, vegetables and pulses were the primary folate source for men but made a lesser contribution to total folate intake. Fruit and berries were only the second most important source for men aged over 64 years, while milk products and bread made a greater contribution to total folate intake in men than in women.

**Table 3.** Folate sources by age group for women (stepwise regression analysis).

18–30 years	% *R*^2^	Change in *R*^2^	31–44 years	% *R*^2^	Change in *R*^2^
Vegetables and pulses	37.8	37.8	Vegetables and pulses	49.1	49.1
Milk products	54.8	17.0	Fruit and berries	63.1	14.0
Bread	62.9	8.0	Bread	70.9	7.8
Fruit and berries	69.9	7.0	Milk products	76.5	5.6
Juice	74.8	4.9	Potatoes	80.2	3.7
Eggs	78.9	4.2	Fast food	83.2	3.0
Fast food	82.6	3.7	Juice	85.6	2.3
Fish and shellfish/seafood	85.3	2.7	Eggs	87.6	2.0
Nuts and seeds	87.3	1.9	Substitute products	89.2	1.6
Potatoes	89.2	1.9	Salads	90.4	1.2
Tea	90.7	1.5	Soups	91.6	1.2
Substitute products	91.6	0.9	Meat	92.6	1.0
Cheese	92.5	0.9	Fish and shellfish/seafood	93.4	0.8
Meat	93.3	0.8	Nuts and sees	94.1	0.7
			Pasta, rice, and grain	94.5	0.4
			Sweet bakery products and sweets	94.7	0.1
**45–64 years**	% *R*^2^	Change in *R*^2^	**>64 years**	% *R*^2^	Change in *R*^2^
Vegetables and pulses	49.7	49.7	Vegetables and pulses	44.7	44.7
Fruit and berries	62.5	12.8	Fruit and berries	61.5	16.9
Bread	70.6	8.1	Juice	70.6	9.1
Milk products	75.2	4.6	Bread	76.6	6.0
Juice	79.4	4.1	Milk products	81.8	5.2
Eggs	82.9	3.5	Potatoes	85.8	4.0
Potatoes	85.5	2.6	Soups	88.6	2.8
Fast food	87.8	2.2	Eggs	90.7	2.1
Tea	89.9	2.1	Nuts and seeds	92.0	1.2
Nuts and seeds	91.1	1.2	Fish and shellfish/seafood	92.8	0.9
Soups	92.3	1.2	Tea	93.7	0.9
Meat	93.5	1.2	Meat	94.6	0.8
Salads	94.7	1.2	Fast food	95.6	1.0
Fish and shellfish/seafood	95.6	0.9	Cheese	96.3	0.7
Cheese	96.1	0.5			
Sweet bakery products and sweets	96.3	0.1			

The cut-off *p* value for entry into the equation was *p* = 0.017 for the 18–30 year age group, *p* = 0.043 for the 31–44 year group, *p* = 0.005 for the 45–64 year group, and *p* = 0.012 for the >64 year age group.

The age was significantly higher for those in the highest (third) folate intake tertile, while the BMI was significantly higher for those in the lowest (first) intake tertile among the men alone. High versus low education was significantly more frequent in the highest folate intake tertile for both genders, while adherence to a vegetarian diet and consumption of organic products were significantly more frequent in the third tertile for the women alone. Smoking was significantly more frequent in the lowest folate intake tertile for both genders (Table 5).

**Table 4.** Folate sources by age group for men (stepwise regression analysis).

18–30 years	% *R*^2^	Change in *R*^2^	31–44 years	% *R*^2^	Change in *R*^2^
Vegetables and pulses	30.3	30.3	Vegetables and pulses	33.9	33.9
Milk products	42.7	13.5	Bread	46.4	12.6
Juice	54.8	12.3	Potatoes	55.1	8.7
Bread	61.6	7.0	Fast food	63.1	8.3
Substitute products	67.3	5.8	Milk products	70.4	6.9
Potatoes	73.1	5.8	Fruit and berries	75.6	5.2
Fast food	78.2	5.1	Juice	80.7	5.0
Eggs	82.5	4.3	Sweet bakery products and sweets	83.7	3.1
Fruit and berries	86.5	4.0	Cereals	86.7	3.0
Meat	88.4	1.9	Eggs	89.0	2.3
Tea	90.3	1.9	Meat	91.3	2.2
Cheese	91.5	1.2	Soups	92.6	1.3
Sweet bakery products and sweets	92.1	0.6	Fish and shellfish/seafood	93.9	1.3
			Salads	95.0	1.1
			Nuts and seeds	95.7	0.7
**45–64 years**	% *R*^2^	Change in *R*^2^	**>64 years**	% *R*^2^	Change in *R*^2^
Vegetables and pulses	44.0	44.0	Vegetables and pulses	37.3	37.3
Bread	57.9	13.9	Fruit and berries	51.4	14.2
Milk products	70.1	12.2	Bread	63.5	12.1
Potatoes	75.5	5.5	Juice	72.0	8.5
Juice	80.4	4.8	Potatoes	79.3	7.2
Fruit and berries	82.8	2.5	Milk products	84.5	5.3
Fast food	85.1	2.3	Fast food	86.5	2.0
Eggs	87.3	2.2	Fish and shellfish/seafood	88.1	1.6
Cereals	89.5	2.2	Nuts and seeds	89.2	1.1
Nuts and seeds	91.3	1.8	Soups	90.5	1.3
Fish and shellfish/seafood	92.8	1.5	Tea	91.5	1.0
Cheese	93.9	1.1	Meat	92.3	0.8
Sauces, dressings, and condiments	94.7	0.8	Cheese	92.9	0.6
Tea	95.4	0.7	Salads	93.2	0.3
Soups	96.0	0.6			
Salads	96.6	0.6			
Pasta, rice and grain	97.0	0.5			
Sweet bakery products and sweets	97.4	0.3			
Fats	97.4	0.1			

The cut-off *p* value for entry into the equation was *p* = 0.011 for the 18–30 year age group, *p* = 0.047 for the 31–44 year group, *p* = 0.015 for the 45–64 year group, and *p* = 0.032 for the >64 year age group.

**Table 5.** Demographic, lifestyle, and dietary factors per tertile (T) of folate intake for men and women.

	Men (*n* = 724)			Women (*n* = 933)		
	T1	T2	T3	T1	T2	T3
	Mean (SD)					
Folate intake (μg/day)^a,^^b^	151.75	227.64	316.46	153.09	224.90	313.91
	(33.70)	(18.87)	(48.62)	(32.84)	(17.80)	(46.53)
Age (years)^a,b^	49.18	52.18	52.22	46.81	47.74	51.29
	(18.06)	(17.00)	(16.49)	(18.04)	(16.32)	(16.78)
Body mass index (kg/m^2^)^a^	26.64	25.99	25.68	25.09	25.27	24.61
	(3.76)	(3.89)	(3.77)	(4.94)	(4.56)	(4.37)
Alcohol intake (g/day)	15.59	19.69	18.70	14.60	14.03	11.85
	(13.64)	(15.26)	(14.64)	(12.12)	(11.05)	(9.81)
	%					
Higher education (university)^c,^^d^	23.6	30.1	46.4	27.5	36.6	35.9
Vegetarian^d^	21.7	21.7	56.5	24.1	25.9	50.0
Diet	45.7	25.7	28.6	38.2	30.9	30.9
Organic fruit and vegetables^d^	29.0	29.7	41.3	30.6	32.7	36.7
Nutritional supplements	26.4	35.6	38.0	32.8	34.0	33.2
Smoking^c,^^d^	45.5	19.6	34.8	51.0	32.9	16.1

ANOVA test: ^a^*p* < 0.05 for men; ^b^*p* < 0.05 for women.

Chi-squared test: ^c^*p* < 0.05 for men; ^d^*p* < 0.05 for women.

Table 6 shows factors related to folate intake (3rd tertile) for the study population. Globally, the probability of reaching the highest tertile of folate intake was higher for men than for women. BMI <25 kg/m^2^ and university education were positively related to folate intake in men. Among women, the probability of reaching the highest tertile of folate intake was higher for over 50-year-olds and for those with high educational level who followed a vegetarian diet, consumed organic products, and did not smoke, and whose alcohol intake was <10 g/day.

**Table 6.** Predictive factors for the highest folate intake (third tertile) for the Riksmaten 2010–11 population.

		%	OR	95% CI			
Gender (all participants)	Women	56.3	Ref.				
	Men		1.69	1.354–2.104			
		Men			Women		
		%	OR	95% CI	%	OR	95% CI
Age	≤Median (50 years)	48.5	Ref.		52.5	Ref.	
	>Median (50 years)		1.28	0.922–1.773		1.67	1.233–2.264
Body mass index	<25 kg/m^2^	44.4	1.52	1.101–2.089	60.6	1.30	0.950–1.778
	≥25 kg/m^2^		Ref.			Ref.	
Educational level	University	38.3	1.60	1.153–2.207	45.7	1.52	1.127–2.059
	Non-university		Ref.			Ref.	
Vegetarian	Yes	3.2	2.20	0.890–5.434	6.1	2.23	1.246–3.988
	No		Ref.			Ref.	
Diet	Yes	4.9	Ref.		5.9	Ref.	
	No		1.52	0.694–3.342		1.10	0.585–2.078
Organic fruit and vegetables	Yes	39.9	1.19	0.854–1.625	44.1	1.55	1.157–2.088
	No		Ref.			Ref.	
Nutritional supplements	Yes	41.3	0.94	0.684–1.302	55.7	1.30	0.964–1.748
	No		Ref.			Ref.	
Smoking	Smoking	15.5	Ref.		16.7	Ref.	
	Non-smoking		1.28	0.819–2.003		2.30	1.431–3.707
Alcohol intake	=Recommended^a^	73.9	0.73	0.514–1.043	71.6	1.42	1.012–1.983
	>Recommended^a^		Ref.			Ref.	

^a^Recommended alcohol intake according to Nordic Nutrition Recommendations [3]: <10 g/day for women and <20 g/day for men.

OR, odds ratio; CI, confidence interval.

## Discussion

This study determined the folate sources of a representative sample of Swedish adults and analyzed the factors that influenced their intake of this vitamin. Vegetables and pulses were found to be the main folate source, and made a higher contribution to total intake in women than in men. Similar results were published by Park et al. [12] in a study across 10 European countries (Denmark, France, Greece, Germany, Italy, The Netherlands, Norway, Spain, Sweden, and the UK), in which vegetables were the first folate source but made a lower percentage contribution (20.8% for men and 25.2% for women) than in the present population. The European study also described differences between the more southerly countries, in which fruit and leafy vegetables were the major vegetable source of folate, and the more northerly countries, in which cabbages and root vegetables were the major vegetable source. Vegetables were the main folate source for pregnant women in Germany, Spain, and Hungary, contributing to 31.2%, 23.4%, and 30.0% of their total folate intake, respectively [23]. However, another study of a sample of young male adults in Norway highlighted that wholegrain bread was the main contributor (around 50%) to total folate intake [24]. More recently, Pounis et al. [25] showed that potatoes and bread were the main sources (contributing to 61.5% of the total folate intake) for participants from Italy, whereas broccoli and root vegetables were the most important contributors (59.1% of total folate intake) for those from the UK.

According to our results in Sweden, other important folate sources were fruit and berries, bread, dairy products, potatoes, and juice. The consumption of fruit and berries was significantly lower in men than in women, in agreement with a previous national food intake survey [26], which may explain why this food group provided less than 5% of the total folate intake for men aged under 65 years but more than 12.8% of the intake for women aged over 30 years. Dairy products were the second folate source for the youngest men and women, which can be especially attributed to their consumption of fermented products such as yogurt [27].

Our analysis of the variables influencing a high intake of folate by this adult population found that gender was a significant factor, with the likelihood of an intake in the highest tertile being 1.69-fold higher for the men than for the women. According to the Riksmaten study [15], men had a higher energy and nutrient intake (except for fiber, vitamin A, and vitamin C) owing to a higher consumption of almost all food groups (Table 2). Among the men, participants with a BMI >25 kg/m^2^ had a lower likelihood of reaching the highest tertile of folate intake than those with lower BMI. Dietary patterns with a high intake of folate-rich foods (vegetables, pulses, cereals, and fruit) have shown a protective effect against the risk of becoming overweight or obese [28].

In the present investigation, men and women with a university education had a higher probability of folate intake in the highest tertile. Current studies [29,30] have shown a more frequent consumption of fruit and vegetables by individuals with a high versus a low educational level. The present participants with a high educational level reported a significantly higher intake of vegetables and pulses than those with a lower educational level (*p* < 0.05), whereas no significant differences were observed for fruit and berry consumption (*p* = 0.054).

The folate intake by women was influenced by other factors, with those aged >50 years having a greater likelihood of being in the highest tertile of folate intake, consistent with previous findings for women in southern Spain [31]. This may be attributable to a closer adherence to traditional dietary patterns, in which there is a greater consumption of vegetables, pulses, fruit, and cereals [32,33]. Various authors have reported a higher folate intake among people who follow vegetarian and vegan diets [34–36], and the folate intake was 30 μg/day higher for vegetarian women (*p* = 0.021) in the present investigation, although no such difference was observed for the men (*p* = 0.123). Furthermore, the likelihood of being in the highest tertile of folate intake was 1.5-fold greater for the women who consumed organic foods, which may be due to a more frequent consumption of plant foods among individuals interested in organically grown food [37].

Smoking and alcohol intake were found to be related to the folate intake of women. Female smokers had a higher risk of being in the lowest tertile of folate intake. Some authors attributed a lower serum folate level in smokers to the interaction of chemical compounds in tobacco with folate and other vitamins (B_6_ and B_12_) [4,38], transforming them into inactive compounds and reducing their availability. Alcohol intake has also been reported to have detrimental effects on folate absorption [5]. The present findings suggest the need to provide additional information on folate-rich foods to young women who smoke and/or consume large amounts of alcohol, although further research is required to explore the relationship of folate intake with tobacco and alcohol consumption.

This study was performed over an entire calendar year and should therefore take account of seasonal variations. It also used a previously tested battery of survey tools and included participants from all parts of Sweden and all types of living environment, including large and small cities as well as rural settings.

However, there was a large number of dropouts from the study, which was completed by only 36% of the initial target population. There were also indications that the intake of fruit and berries may have been lower in non-respondents than in respondents. The food database used also has some shortcomings and does not take account of variations in folate content according to season, storage, and cooking. Hence, these results are only indicative of the highest intakes, and it should also be borne in mind that the issue of bioavailability is not considered.

In conclusion, this study of the folate intake of Swedish adults, based on the Riksmaten survey in 2010–11, identifies vegetables and pulses as the most important folate source. Data obtained on factors related to folate consumption, including gender, age, lifestyle, and dietary habits, may provide useful background information for the development of specific nutrition education programs to increase the intake of this vitamin in high-risk groups.
